# 

**Published:** 1879-05-20

**Authors:** 


### TABLE OF CONTENTS.

ORIGINAL AND SELECTED ARTICLES.

On the Treatment of Scarlatina, by T. R. Greenley, M.D., of Kentucky, 161. Vomiting of Pregnancy—Treatment with Wine of Ipecac, by D. B. Nesbit, M. D., of Georgia, 163; Whisky Hypodermically in Collapse from Loss of Blood, by W. N. Ames, M. D., of Miss., 164. Chloroform in Convulsions of Infants and Children, by J. D. Blake, M. D., 165. Case of Cephalic Version by External Manipulation, 168. Interesting Case of Vomiting, by Prof. Da Costa, 170 An Improvement in the Treatment of Epididymitis, 172. Carbolized Jute as a Wound Dressing, by Robert F. Weir, M. D., 174. Oxalate of Cerium in Pregnancy, by Dr. F. E. Image, 176. Cupping in Anthrax, 176. Buffalo Lithia Springs, by Dr. Goodbridge A. Wilson, 177.

ABSTRACTS AND GLEANINGS.

Water and Air from Yellow Fever Rooms, 181. Morphia Hypodermically, 182. Female Catheterism, 183 Cold and Elevation as Hæmostatics, 184. Treatment of Diphtheria, 184. Chlorine in Scarlet Fever and Diphtheria, 185. Gelseminum for Hectic, 186. Potato and Diphtheria, 187. Prolapse of the Rectum in Infants, 187. Thermometer in Abdominal Affections, 188. Alkalies in Dyspepsia, 188. Treatment of Burns, 189. Intussusception in Infants, 189. Headache, 189. Digitalis in Suppression of Urine, 190. Silicylic Acid Enemata in Dysentry; Cure for Hiccough; Salicylate of Soda in Rheumatism; Bromide of Potassium in Coughs; Guaco in Cancer, 190.

SCIENTIFIC ITEMS.

Is Science Benevolent? 191. New Crater in the Moon; Electric Gas Lighting Apparatus, 191. Opium Smoking; Cost of the Yellow Fever; Air Purifier; New Anæsthetic; Astronomical Observations; Trichinæ destroyed by Sulphurous Acid, 192.

PRACTICAL NOTES AND FORMULÆ.

Mild Zinc Ointment; Gentian and Rhubarb Draught; Alkaline Columbo Draught; Hair Dye without Lead or Silver; Oxide of Zinc, 193. Chloral as a Counter-Irritant; Cough Mixture for Consumption; Dr. Kitchener's Peristaltic Persuaders; In Chronic Bronchitis; For Freckles; To Restrain Excessive Secretion, and in Hemorrhage, 194. Toothache Remedy; In Syphilitic Skin Diseases, Cachexia, etc.; Laxative Tonic Pills; In Irritable Stomach of Young Children; New Hypnotic; Useful Aperient; Capsicum with Quinia; Liquor Arsenicalis in Prickly Heat, 195.

EDITORIAL AND MISCELLANEOUS.

Professional Fraternity, 196. Association of American Medical Editors, 197. American Medical Association, 198. Editorial Dinner, 199. Medical Association of Georgia; Special Notices, 200.

